# 2-[2-(5-Bromo­thio­phen-2-yl)-4,5-diphenyl-1*H*-imidazol-1-yl]-3-phenyl­propan-1-ol

**DOI:** 10.1107/S1600536813021016

**Published:** 2013-08-03

**Authors:** Jie Gao, Liangru Yang, Wenpeng Mai, Jinwei Yuan, Pu Mao

**Affiliations:** a School of Chemistry and Chemical Engineering, Henan University of Technology, Zhengzhou 450001, People’s Republic of China

## Abstract

In the title compound, C_28_H_23_BrN_2_OS, the dihedral angles formed by the imidazole ring with the 5-bromo­thio­phenyl and phenyl rings are 76.90 (8), 34.02 (10) and 80.93 (11)°, respectively. The chiral centre maintains the *S* configuration of the l-phenyl­alaninol starting material. In the crystal, mol­ecules are linked by O—H⋯N hydrogen bonds, forming chains running parallel to the *a-*axis direction.

## Related literature

For the synthesis of imidazole rings, see: Jiang *et al.* (2009 ▶); Wu *et al.* (2010 ▶); Eseola *et al.* (2010 ▶). For related compounds synthesized by our group, see: Mao *et al.* (2010 ▶); Yang *et al.* (2012 ▶); Xiao *et al.* (2012 ▶).
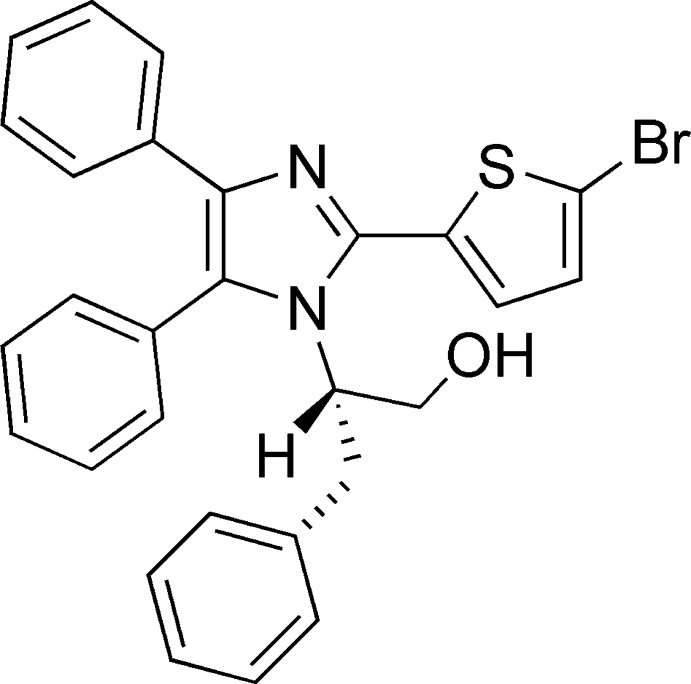


## Experimental

### 

#### Crystal data

C_28_H_23_BrN_2_OS
*M* *_r_* = 515.45
Orthorhombic, 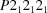

*a* = 9.36677 (18) Å
*b* = 15.8434 (3) Å
*c* = 16.1452 (3) Å
*V* = 2395.97 (8) Å^3^
*Z* = 4
Cu *K*α radiation
μ = 3.33 mm^−1^
*T* = 291 K
0.3 × 0.28 × 0.26 mm

#### Data collection

Agilent Xcalibur (Eos, Gemini) diffractometer
Absorption correction: multi-scan (*CrysAlis PRO*; Agilent, 2011 ▶) *T*_min_ = 0.853, *T*_max_ = 1.000
8866 measured reflections
4264 independent reflections
4033 reflections with *I* > 2σ(*I*)
*R*_int_ = 0.019

#### Refinement

*R*[*F* ^2^ > 2σ(*F* ^2^)] = 0.033
*wR*(*F* ^2^) = 0.085
*S* = 1.03
4264 reflections
302 parameters
H atoms treated by a mixture of independent and constrained refinement
Δρ_max_ = 0.22 e Å^−3^
Δρ_min_ = −0.44 e Å^−3^
Absolute structure: Flack (1983 ▶); 1834 Friedel pairs
Absolute structure parameter: −0.004 (16)

### 

Data collection: *CrysAlis PRO* (Agilent, 2011 ▶); cell refinement: *CrysAlis PRO*; data reduction: *CrysAlis PRO*; program(s) used to solve structure: *SHELXS97* (Sheldrick, 2008 ▶); program(s) used to refine structure: *SHELXL97* (Sheldrick, 2008 ▶); molecular graphics: *OLEX2* (Dolomanov *et al.*, 2009 ▶); software used to prepare material for publication: *OLEX2*.

## Supplementary Material

Crystal structure: contains datablock(s) I, global. DOI: 10.1107/S1600536813021016/rz5079sup1.cif

Structure factors: contains datablock(s) I. DOI: 10.1107/S1600536813021016/rz5079Isup2.hkl

Supplementary material file. DOI: 10.1107/S1600536813021016/rz5079Isup3.cml

Additional supplementary materials:  crystallographic information; 3D view; checkCIF report

## Figures and Tables

**Table 1:** Hydrogen-bond geometry (Å, °)

*D*—H⋯*A*	*D*—H	H⋯*A*	*D*⋯*A*	*D*—H⋯*A*
O1—H1⋯N1^i^	0.83 (4)	2.04 (4)	2.838 (3)	162 (4)

Symmetry code: (i) 
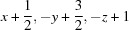
.

**Fig. 1.**
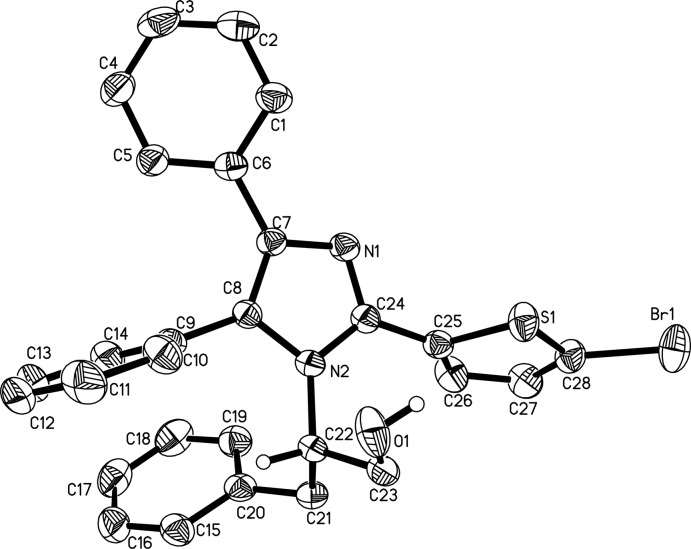
The molecular structure of the title compound showing 30% probability displacement ellipsoids. Aromatic, methylene and methyne hydrogen atoms are omitted for clarity.

## supplementary crystallographic information

### 1. Comment

The development of imidazoles with an heterocyclic substituent in 2-position from readily available inexpensive starting materials has been an active topic in modern organic chemistry (Jiang *et al.*, 2009; Wu *et al.*, 2010; Eseola *et al.*, 2010). Our group is interested in the research of chiral imidazolium derivatives derived from natural amino acids (Mao *et al.*, 2010; Yang *et al.*, 2012; Xiao *et al.*, 2012). A convenient and highly efficient one-pot-multicomponent protocol has been developed for the synthesis of the title compound from *L*-phenylalaninol, 5-bromothiophene-2-carbaldehyde, dibenzoyl and ammonium acetate.

The molecular structure of the title compound is shown in Figure 1. As expected, the imidazole core (C7/C8/N2/C24/N1) is essentially planar, the maximum deviation being 0.008 (3) Å for atom C24. The dihedral angle between the 5-bromothiophenyl ring and imidazole ring is 76.90 (8)°. The dihedral angles between the two phenyl substituents (C1–C6, C9–C14) and the imidazole ring are 34.02 (10)° and 80.93 (11)°, respectively. The chiral C22 carbon atom maintains the *S* configuration of the *L*-phenylalaninol starting material. In the crystal, intermolecular O—H···N hydrogen bonds (Table 1) link molecules into chains running parallel to the *a* axis.

### 2. Experimental

The starting materials, *L*-phenylalaninol, benzil, ammonium acetate and 5-bromothiophene-2-carbaldehyde, are commercially available. In a three-neck round-bottomed flask fitted with a reflux condenser, *L*-phenylalaninol (0.76 g, 5 mmol), molar equivalents benzil, ammonium acetate and 5-bromothiophene-2-carbaldehyde were dissolved in CH_3_OH (30 mL). The mixture was kept at 65°C for 12 h. The resulting solution was cooled to room temperature and evaporation of the solvent gave the crude product. Crystallization of the crude product in CH_3_OH afforded colourless crystals of the title compound.

### 3. Refinement

The hydroxy H atom was located in a difference Fourier map and refined freely. All other H atoms were placed geometrically and refined as riding, with C—H = 0.93-0.98 Å, and with *U*_iso_(H) = 1.2 *U*_eq_(C).

### Crystal data

**Table:**

C_28_H_23_BrN_2_OS	*F*(000) = 1056
*M**_r_* = 515.45	*D*_x_ = 1.429 Mg m^−^^3^
Orthorhombic, *P*2_1_2_1_2_1_	Cu *K*α radiation, λ = 1.5418 Å
Hall symbol: P 2ac 2ab	Cell parameters from 4493 reflections
*a* = 9.36677 (18) Å	θ = 3.9–72.3°
*b* = 15.8434 (3) Å	µ = 3.33 mm^−^^1^
*c* = 16.1452 (3) Å	*T* = 291 K
*V* = 2395.97 (8) Å^3^	Block, colourless
*Z* = 4	0.3 × 0.28 × 0.26 mm

### Data collection

**Table:**

Agilent Xcalibur (Eos, Gemini) diffractometer	4264 independent reflections
Radiation source: Enhance (Cu) X-ray Source	4033 reflections with *I* > 2σ(*I*)
Graphite monochromator	*R*_int_ = 0.019
Detector resolution: 16.2312 pixels mm^-1^	θ_max_ = 67.1°, θ_min_ = 3.9°
ω scans	*h* = −11→7
Absorption correction: multi-scan (*CrysAlis PRO*; Agilent, 2011)	*k* = −18→18
*T*_min_ = 0.853, *T*_max_ = 1.000	*l* = −19→19
8866 measured reflections	

### Refinement

**Table:**

Refinement on *F*^2^	Secondary atom site location: difference Fourier map
Least-squares matrix: full	Hydrogen site location: inferred from neighbouring sites
*R*[*F*^2^ > 2σ(*F*^2^)] = 0.033	H atoms treated by a mixture of independent and constrained refinement
*wR*(*F*^2^) = 0.085	*w* = 1/[σ^2^(*F*_o_^2^) + (0.0429*P*)^2^ + 0.2535*P*] where *P* = (*F*_o_^2^ + 2*F*_c_^2^)/3
*S* = 1.03	(Δ/σ)_max_ = 0.001
4264 reflections	Δρ_max_ = 0.22 e Å^−^^3^
302 parameters	Δρ_min_ = −0.44 e Å^−^^3^
0 restraints	Absolute structure: Flack (1983); 1834 Friedel pairs
Primary atom site location: structure-invariant direct methods	Absolute structure parameter: −0.004 (16)

### Special details

**Table:**

Geometry. All e.s.d.'s (except the e.s.d. in the dihedral angle between two l.s. planes) are estimated using the full covariance matrix. The cell e.s.d.'s are taken into account individually in the estimation of e.s.d.'s in distances, angles and torsion angles; correlations between e.s.d.'s in cell parameters are only used when they are defined by crystal symmetry. An approximate (isotropic) treatment of cell e.s.d.'s is used for estimating e.s.d.'s involving l.s. planes.
Refinement. Refinement of *F*^2^ against ALL reflections. The weighted *R*-factor *wR* and goodness of fit *S* are based on *F*^2^, conventional *R*-factors *R* are based on *F*, with *F* set to zero for negative *F*^2^. The threshold expression of *F*^2^ > σ(*F*^2^) is used only for calculating *R*-factors(gt) *etc*. and is not relevant to the choice of reflections for refinement. *R*-factors based on *F*^2^ are statistically about twice as large as those based on *F*, and *R*- factors based on ALL data will be even larger.

### Fractional atomic coordinates and isotropic or equivalent isotropic displacement parameters (Å^2^)

**Table:**

	*x*	*y*	*z*	*U*_iso_*/*U*_eq_	
Br1	0.26010 (5)	0.97884 (3)	0.26215 (3)	0.08774 (14)	
S1	0.29866 (8)	0.83956 (5)	0.39570 (5)	0.06109 (18)	
O1	0.5090 (3)	0.60857 (15)	0.42808 (18)	0.0755 (7)	
H1	0.537 (4)	0.658 (3)	0.428 (3)	0.093 (14)*	
N1	0.1628 (2)	0.73730 (13)	0.56318 (13)	0.0467 (4)	
N2	0.2202 (2)	0.62572 (12)	0.48784 (12)	0.0422 (4)	
C1	0.1975 (3)	0.75939 (19)	0.74105 (18)	0.0607 (6)	
H1A	0.2348	0.8022	0.7082	0.073*	
C2	0.1762 (4)	0.7731 (2)	0.8247 (2)	0.0731 (9)	
H2	0.2011	0.8246	0.8481	0.088*	
C3	0.1178 (4)	0.7098 (2)	0.87404 (18)	0.0737 (9)	
H3	0.1026	0.7192	0.9302	0.088*	
C4	0.0831 (4)	0.6345 (2)	0.83955 (19)	0.0667 (8)	
H4	0.0435	0.5923	0.8723	0.080*	
C5	0.1059 (3)	0.61977 (17)	0.75615 (17)	0.0561 (6)	
H5	0.0825	0.5675	0.7337	0.067*	
C6	0.1632 (3)	0.68165 (16)	0.70573 (15)	0.0464 (5)	
C7	0.1817 (2)	0.67005 (15)	0.61521 (14)	0.0429 (5)	
C8	0.2177 (2)	0.59945 (14)	0.57013 (14)	0.0412 (5)	
C9	0.2546 (3)	0.51201 (13)	0.59565 (14)	0.0445 (5)	
C10	0.3964 (3)	0.48806 (18)	0.6020 (2)	0.0628 (7)	
H10	0.4682	0.5264	0.5892	0.075*	
C11	0.4316 (5)	0.4072 (2)	0.6272 (3)	0.0819 (11)	
H11	0.5269	0.3913	0.6313	0.098*	
C12	0.3253 (5)	0.3500 (2)	0.6463 (2)	0.0811 (11)	
H12	0.3488	0.2959	0.6641	0.097*	
C13	0.1856 (5)	0.3734 (2)	0.6388 (2)	0.0772 (10)	
H13	0.1139	0.3348	0.6512	0.093*	
C14	0.1495 (3)	0.45393 (19)	0.61287 (19)	0.0601 (7)	
H14	0.0539	0.4688	0.6071	0.072*	
C15	0.0418 (3)	0.41288 (18)	0.4051 (2)	0.0625 (7)	
H15	0.1258	0.3886	0.3856	0.075*	
C16	−0.0611 (4)	0.3619 (2)	0.4394 (2)	0.0754 (9)	
H16	−0.0465	0.3040	0.4427	0.091*	
C17	−0.1868 (4)	0.3970 (3)	0.4690 (2)	0.0799 (10)	
H17	−0.2571	0.3627	0.4919	0.096*	
C18	−0.2062 (4)	0.4825 (3)	0.4643 (2)	0.0789 (9)	
H18	−0.2897	0.5067	0.4845	0.095*	
C19	−0.1017 (3)	0.5331 (2)	0.42947 (19)	0.0636 (7)	
H19	−0.1162	0.5911	0.4267	0.076*	
C20	0.0242 (3)	0.49927 (17)	0.39859 (16)	0.0512 (6)	
C21	0.1378 (3)	0.55276 (18)	0.35912 (15)	0.0514 (6)	
H21A	0.0956	0.6056	0.3412	0.062*	
H21B	0.1737	0.5240	0.3104	0.062*	
C22	0.2629 (3)	0.57177 (13)	0.41736 (14)	0.0440 (5)	
H22	0.2925	0.5176	0.4410	0.053*	
C23	0.3935 (3)	0.60763 (16)	0.37267 (17)	0.0515 (6)	
H23A	0.4164	0.5730	0.3250	0.062*	
H23B	0.3736	0.6645	0.3535	0.062*	
C24	0.1849 (3)	0.70913 (15)	0.48779 (15)	0.0440 (5)	
C25	0.1707 (3)	0.76397 (15)	0.41474 (15)	0.0479 (5)	
C26	0.0587 (4)	0.7737 (2)	0.3627 (2)	0.0661 (8)	
H26	−0.0214	0.7391	0.3639	0.079*	
C27	0.0743 (4)	0.8412 (2)	0.3063 (2)	0.0672 (8)	
H27	0.0069	0.8558	0.2665	0.081*	
C28	0.1975 (3)	0.88144 (18)	0.31747 (17)	0.0582 (7)	

### Atomic displacement parameters (Å^2^)

**Table:**

	*U*^11^	*U*^22^	*U*^33^	*U*^12^	*U*^13^	*U*^23^
Br1	0.0876 (2)	0.0822 (2)	0.0934 (3)	0.00601 (19)	0.0010 (2)	0.04352 (19)
S1	0.0579 (4)	0.0608 (4)	0.0646 (4)	−0.0061 (3)	−0.0146 (3)	0.0179 (3)
O1	0.0605 (12)	0.0598 (12)	0.1062 (19)	−0.0204 (10)	−0.0170 (12)	0.0204 (13)
N1	0.0503 (11)	0.0417 (10)	0.0482 (10)	0.0091 (9)	0.0002 (9)	−0.0031 (8)
N2	0.0445 (10)	0.0396 (9)	0.0424 (9)	0.0005 (8)	−0.0027 (8)	−0.0058 (7)
C1	0.0658 (15)	0.0574 (14)	0.0588 (15)	0.0018 (13)	0.0066 (13)	−0.0128 (13)
C2	0.082 (2)	0.0712 (19)	0.0659 (18)	0.0060 (17)	0.0016 (16)	−0.0252 (16)
C3	0.089 (2)	0.086 (2)	0.0468 (14)	0.0267 (19)	0.0031 (15)	−0.0161 (15)
C4	0.081 (2)	0.0697 (19)	0.0492 (15)	0.0164 (16)	0.0028 (15)	0.0049 (14)
C5	0.0658 (15)	0.0532 (14)	0.0493 (14)	0.0095 (12)	0.0009 (13)	−0.0003 (12)
C6	0.0430 (12)	0.0497 (13)	0.0465 (12)	0.0116 (10)	−0.0045 (10)	−0.0066 (10)
C7	0.0429 (11)	0.0438 (11)	0.0419 (11)	0.0059 (10)	0.0007 (9)	−0.0027 (10)
C8	0.0394 (12)	0.0403 (10)	0.0438 (11)	−0.0012 (9)	−0.0037 (9)	−0.0025 (9)
C9	0.0522 (12)	0.0398 (10)	0.0416 (10)	0.0021 (10)	−0.0035 (10)	−0.0058 (8)
C10	0.0583 (16)	0.0536 (15)	0.0765 (18)	0.0071 (13)	−0.0085 (15)	−0.0030 (15)
C11	0.085 (2)	0.070 (2)	0.092 (3)	0.0318 (19)	−0.016 (2)	−0.0027 (19)
C12	0.135 (3)	0.0417 (14)	0.0667 (19)	0.0170 (19)	−0.007 (2)	0.0008 (14)
C13	0.112 (3)	0.0451 (15)	0.074 (2)	−0.0154 (17)	0.003 (2)	0.0015 (14)
C14	0.0639 (17)	0.0530 (14)	0.0633 (16)	−0.0069 (13)	0.0005 (14)	−0.0037 (13)
C15	0.0645 (17)	0.0576 (16)	0.0654 (17)	−0.0092 (13)	−0.0040 (14)	−0.0081 (14)
C16	0.093 (2)	0.0605 (18)	0.073 (2)	−0.0212 (17)	−0.0041 (19)	0.0017 (15)
C17	0.075 (2)	0.099 (3)	0.0658 (19)	−0.035 (2)	−0.0058 (17)	0.0093 (18)
C18	0.0549 (17)	0.110 (3)	0.0721 (19)	−0.0020 (18)	0.0054 (14)	0.0033 (19)
C19	0.0636 (17)	0.0641 (17)	0.0633 (16)	0.0032 (14)	−0.0007 (14)	−0.0025 (14)
C20	0.0532 (14)	0.0564 (14)	0.0441 (12)	−0.0059 (11)	−0.0097 (11)	−0.0081 (11)
C21	0.0592 (15)	0.0526 (14)	0.0426 (12)	−0.0050 (12)	−0.0050 (11)	−0.0059 (11)
C22	0.0501 (12)	0.0370 (9)	0.0451 (11)	−0.0024 (10)	0.0006 (11)	−0.0057 (8)
C23	0.0566 (15)	0.0424 (12)	0.0556 (14)	−0.0028 (11)	0.0051 (12)	−0.0070 (11)
C24	0.0435 (12)	0.0419 (11)	0.0466 (12)	0.0026 (10)	−0.0036 (10)	−0.0005 (10)
C25	0.0509 (13)	0.0442 (12)	0.0486 (12)	0.0049 (10)	−0.0007 (11)	−0.0029 (10)
C26	0.0626 (17)	0.0566 (15)	0.079 (2)	−0.0013 (13)	−0.0202 (16)	0.0057 (15)
C27	0.0716 (19)	0.0643 (17)	0.0658 (18)	0.0079 (16)	−0.0233 (15)	0.0063 (15)
C28	0.0672 (17)	0.0532 (14)	0.0544 (14)	0.0103 (13)	−0.0025 (13)	0.0117 (12)

### Geometric parameters (Å, º)

**Table:**

Br1—C28	1.877 (3)	C12—H12	0.9300
S1—C25	1.722 (3)	C12—C13	1.366 (6)
S1—C28	1.713 (3)	C13—H13	0.9300
O1—H1	0.83 (4)	C13—C14	1.385 (5)
O1—C23	1.404 (4)	C14—H14	0.9300
N1—C7	1.368 (3)	C15—H15	0.9300
N1—C24	1.313 (3)	C15—C16	1.374 (5)
N2—C8	1.392 (3)	C15—C20	1.383 (4)
N2—C22	1.478 (3)	C16—H16	0.9300
N2—C24	1.362 (3)	C16—C17	1.386 (6)
C1—H1A	0.9300	C17—H17	0.9300
C1—C2	1.383 (4)	C17—C18	1.370 (5)
C1—C6	1.395 (4)	C18—H18	0.9300
C2—H2	0.9300	C18—C19	1.383 (5)
C2—C3	1.392 (5)	C19—H19	0.9300
C3—H3	0.9300	C19—C20	1.388 (4)
C3—C4	1.357 (5)	C20—C21	1.502 (4)
C4—H4	0.9300	C21—H21A	0.9700
C4—C5	1.383 (4)	C21—H21B	0.9700
C5—H5	0.9300	C21—C22	1.532 (3)
C5—C6	1.382 (4)	C22—H22	0.9800
C6—C7	1.483 (3)	C22—C23	1.530 (4)
C7—C8	1.377 (3)	C23—H23A	0.9700
C8—C9	1.486 (3)	C23—H23B	0.9700
C9—C10	1.385 (4)	C24—C25	1.471 (3)
C9—C14	1.376 (4)	C25—C26	1.352 (4)
C10—H10	0.9300	C26—H26	0.9300
C10—C11	1.385 (4)	C26—C27	1.412 (4)
C11—H11	0.9300	C27—H27	0.9300
C11—C12	1.381 (6)	C27—C28	1.331 (5)
			
C28—S1—C25	90.92 (14)	C20—C15—H15	119.1
C23—O1—H1	105 (3)	C15—C16—H16	120.0
C24—N1—C7	106.5 (2)	C15—C16—C17	120.0 (3)
C8—N2—C22	124.47 (19)	C17—C16—H16	120.0
C24—N2—C8	106.65 (19)	C16—C17—H17	120.4
C24—N2—C22	128.8 (2)	C18—C17—C16	119.3 (3)
C2—C1—H1A	119.8	C18—C17—H17	120.4
C2—C1—C6	120.3 (3)	C17—C18—H18	119.9
C6—C1—H1A	119.8	C17—C18—C19	120.1 (3)
C1—C2—H2	119.9	C19—C18—H18	119.9
C1—C2—C3	120.2 (3)	C18—C19—H19	119.2
C3—C2—H2	119.9	C18—C19—C20	121.6 (3)
C2—C3—H3	120.2	C20—C19—H19	119.2
C4—C3—C2	119.5 (3)	C15—C20—C19	117.2 (3)
C4—C3—H3	120.2	C15—C20—C21	120.4 (3)
C3—C4—H4	119.6	C19—C20—C21	122.5 (3)
C3—C4—C5	120.7 (3)	C20—C21—H21A	109.0
C5—C4—H4	119.6	C20—C21—H21B	109.0
C4—C5—H5	119.6	C20—C21—C22	113.1 (2)
C6—C5—C4	120.9 (3)	H21A—C21—H21B	107.8
C6—C5—H5	119.6	C22—C21—H21A	109.0
C1—C6—C7	119.0 (3)	C22—C21—H21B	109.0
C5—C6—C1	118.4 (3)	N2—C22—C21	112.3 (2)
C5—C6—C7	122.5 (2)	N2—C22—H22	106.5
N1—C7—C6	119.6 (2)	N2—C22—C23	111.38 (19)
N1—C7—C8	109.9 (2)	C21—C22—H22	106.5
C8—C7—C6	130.6 (2)	C23—C22—C21	113.3 (2)
N2—C8—C9	122.65 (19)	C23—C22—H22	106.5
C7—C8—N2	105.4 (2)	O1—C23—C22	108.6 (2)
C7—C8—C9	131.9 (2)	O1—C23—H23A	110.0
C10—C9—C8	119.9 (2)	O1—C23—H23B	110.0
C14—C9—C8	120.9 (2)	C22—C23—H23A	110.0
C14—C9—C10	119.2 (3)	C22—C23—H23B	110.0
C9—C10—H10	119.9	H23A—C23—H23B	108.3
C11—C10—C9	120.2 (3)	N1—C24—N2	111.6 (2)
C11—C10—H10	119.9	N1—C24—C25	121.9 (2)
C10—C11—H11	120.0	N2—C24—C25	126.5 (2)
C12—C11—C10	120.1 (3)	C24—C25—S1	119.39 (19)
C12—C11—H11	120.0	C26—C25—S1	110.4 (2)
C11—C12—H12	120.2	C26—C25—C24	129.5 (3)
C13—C12—C11	119.5 (3)	C25—C26—H26	123.0
C13—C12—H12	120.2	C25—C26—C27	114.0 (3)
C12—C13—H13	119.6	C27—C26—H26	123.0
C12—C13—C14	120.7 (3)	C26—C27—H27	124.3
C14—C13—H13	119.6	C28—C27—C26	111.4 (3)
C9—C14—C13	120.2 (3)	C28—C27—H27	124.3
C9—C14—H14	119.9	S1—C28—Br1	119.80 (18)
C13—C14—H14	119.9	C27—C28—Br1	126.9 (2)
C16—C15—H15	119.1	C27—C28—S1	113.2 (2)
C16—C15—C20	121.9 (3)		
			
S1—C25—C26—C27	0.6 (4)	C10—C11—C12—C13	1.0 (6)
N1—C7—C8—N2	−0.1 (3)	C11—C12—C13—C14	−0.5 (6)
N1—C7—C8—C9	177.5 (2)	C12—C13—C14—C9	−1.1 (5)
N1—C24—C25—S1	72.7 (3)	C14—C9—C10—C11	−1.5 (5)
N1—C24—C25—C26	−96.9 (4)	C15—C16—C17—C18	−0.5 (5)
N2—C8—C9—C10	79.5 (3)	C15—C20—C21—C22	−79.7 (3)
N2—C8—C9—C14	−100.2 (3)	C16—C15—C20—C19	0.9 (5)
N2—C22—C23—O1	62.7 (3)	C16—C15—C20—C21	−178.7 (3)
N2—C24—C25—S1	−107.7 (3)	C16—C17—C18—C19	0.6 (5)
N2—C24—C25—C26	82.7 (4)	C17—C18—C19—C20	0.1 (5)
C1—C2—C3—C4	0.6 (6)	C18—C19—C20—C15	−0.9 (4)
C1—C6—C7—N1	−31.8 (4)	C18—C19—C20—C21	178.8 (3)
C1—C6—C7—C8	147.7 (3)	C19—C20—C21—C22	100.6 (3)
C2—C1—C6—C5	1.0 (4)	C20—C15—C16—C17	−0.3 (5)
C2—C1—C6—C7	177.5 (3)	C20—C21—C22—N2	−66.4 (3)
C2—C3—C4—C5	0.4 (5)	C20—C21—C22—C23	166.3 (2)
C3—C4—C5—C6	−0.7 (5)	C21—C22—C23—O1	−169.6 (2)
C4—C5—C6—C1	0.0 (4)	C22—N2—C8—C7	176.0 (2)
C4—C5—C6—C7	−176.4 (3)	C22—N2—C8—C9	−1.8 (4)
C5—C6—C7—N1	144.6 (3)	C22—N2—C24—N1	−175.4 (2)
C5—C6—C7—C8	−35.8 (4)	C22—N2—C24—C25	5.0 (4)
C6—C1—C2—C3	−1.3 (5)	C24—N1—C7—C6	−179.7 (2)
C6—C7—C8—N2	−179.6 (2)	C24—N1—C7—C8	0.6 (3)
C6—C7—C8—C9	−2.1 (5)	C24—N2—C8—C7	−0.5 (3)
C7—N1—C24—N2	−1.0 (3)	C24—N2—C8—C9	−178.3 (2)
C7—N1—C24—C25	178.6 (2)	C24—N2—C22—C21	−71.2 (3)
C7—C8—C9—C10	−97.7 (3)	C24—N2—C22—C23	57.1 (3)
C7—C8—C9—C14	82.6 (3)	C24—C25—C26—C27	170.9 (3)
C8—N2—C22—C21	113.1 (2)	C25—S1—C28—Br1	176.47 (18)
C8—N2—C22—C23	−118.7 (2)	C25—S1—C28—C27	0.2 (3)
C8—N2—C24—N1	1.0 (3)	C25—C26—C27—C28	−0.4 (4)
C8—N2—C24—C25	−178.6 (2)	C26—C27—C28—Br1	−175.9 (2)
C8—C9—C10—C11	178.8 (3)	C26—C27—C28—S1	0.1 (4)
C8—C9—C14—C13	−178.2 (3)	C28—S1—C25—C24	−171.9 (2)
C9—C10—C11—C12	−0.1 (6)	C28—S1—C25—C26	−0.5 (2)
C10—C9—C14—C13	2.1 (4)		

### Hydrogen-bond geometry (Å, º)

**Table:**

*D*—H···*A*	*D*—H	H···*A*	*D*···*A*	*D*—H···*A*
O1—H1···N1^i^	0.83 (4)	2.04 (4)	2.838 (3)	162 (4)

Symmetry code: (i) *x*+1/2, −*y*+3/2, −*z*+1.
